# AutoProstate: Towards Automated Reporting of Prostate MRI for Prostate Cancer Assessment Using Deep Learning

**DOI:** 10.3390/cancers13236138

**Published:** 2021-12-06

**Authors:** Pritesh Mehta, Michela Antonelli, Saurabh Singh, Natalia Grondecka, Edward W. Johnston, Hashim U. Ahmed, Mark Emberton, Shonit Punwani, Sébastien Ourselin

**Affiliations:** 1 Department of Medical Physics and Biomedical Engineering, University College London, London WC1E 6BT, UK; 2 School of Biomedical Engineering Imaging Sciences, King’s College London, London SE1 7EH, UK; michela.antonelli@kcl.ac.uk (M.A.); sebastien.ourselin@kcl.ac.uk (S.O.); 3 Centre for Medical Imaging, University College London, London WC1E 6BT, UK; saurabh.singh@ucl.ac.uk (S.S.); s.punwani@ucl.ac.uk (S.P.); 4 Department of Medical Radiology, Medical University of Lublin, 20-059 Lublin, Poland; ngrondecka@wp.pl; 5 Interventional Radiology, Royal Marsden Hospital, London SW3 6JJ, UK; edward.johnston@ucl.ac.uk; 6 Imperial Prostate, Department of Surgery and Cancer, Faculty of Medicine, Imperial College London, London SW7 2AZ, UK; hashim.ahmed@imperial.ac.uk; 7 Division of Surgery and Interventional Science, Faculty of Medical Sciences, University College London, London WC1E 6BT, UK; m.emberton@ucl.ac.uk

**Keywords:** automatic report, computer-aided diagnosis, convolutional neural network, deep learning, lesion detection, lesion classification, magnetic resonance imaging, prostate cancer, segmentation

## Abstract

**Simple Summary:**

International guidelines recommend multiparametric magnetic resonance imaging (mpMRI) of the prostate for use by radiologists to identify lesions containing clinically significant prostate cancer, prior to confirmatory biopsy. Automatic assessment of prostate mpMRI using artificial intelligence algorithms holds a currently unrealized potential to improve the diagnostic accuracy achievable by radiologists alone, improve the reporting consistency between radiologists, and enhance reporting quality. In this work, we introduce AutoProstate: a deep learning-powered framework for automatic MRI-based prostate cancer assessment. In particular, AutoProstate utilizes patient data and biparametric MRI to populate an automatic web-based report which includes segmentations of the whole prostate, prostatic zones, and candidate clinically significant prostate cancer lesions, and in addition, several derived characteristics with clinical value are presented. Notably, AutoProstate performed well in external validation using the PICTURE study dataset, suggesting value in prospective multicentre validation, with a view towards future deployment into the prostate cancer diagnostic pathway.

**Abstract:**

Multiparametric magnetic resonance imaging (mpMRI) of the prostate is used by radiologists to identify, score, and stage abnormalities that may correspond to clinically significant prostate cancer (CSPCa). Automatic assessment of prostate mpMRI using artificial intelligence algorithms may facilitate a reduction in missed cancers and unnecessary biopsies, an increase in inter-observer agreement between radiologists, and an improvement in reporting quality. In this work, we introduce AutoProstate, a deep learning-powered framework for automatic MRI-based prostate cancer assessment. AutoProstate comprises of three modules: Zone-Segmenter, CSPCa-Segmenter, and Report-Generator. Zone-Segmenter segments the prostatic zones on T2-weighted imaging, CSPCa-Segmenter detects and segments CSPCa lesions using biparametric MRI, and Report-Generator generates an automatic web-based report containing four sections: *Patient Details*, *Prostate Size and PSA Density*, *Clinically Significant Lesion Candidates*, and *Findings Summary*. In our experiment, AutoProstate was trained using the publicly available PROSTATEx dataset, and externally validated using the PICTURE dataset. Moreover, the performance of AutoProstate was compared to the performance of an experienced radiologist who prospectively read PICTURE dataset cases. In comparison to the radiologist, AutoProstate showed statistically significant improvements in prostate volume and prostate-specific antigen density estimation. Furthermore, AutoProstate matched the CSPCa lesion detection sensitivity of the radiologist, which is paramount, but produced more false positive detections.

## 1. Introduction

Radiologists use prostate multiparametric magnetic resonance imaging (mpMRI) to detect, score, and stage lesions that may correspond to clinically significant prostate cancer (CSPCa), whose status can later be confirmed using MR-guided targeted biopsy and histopathological grading [1]. However, the current diagnostic approach must be improved to reduce the small proportion of men with CSPCa who are missed by mpMRI, to reduce the large number of men who undergo unnecessary biopsies, and to increase the inter-observer agreement between readers [2]. In addition to lesion assessment, radiologists use prostate mpMRI to estimate prostate volume using the ellipsoid formula [3]. Primarily, prostate volume is required for calculating prostate-specific antigen density (PSAd), which has been shown to be a predictor of CSPCa [4]. However, the ellipsoid formula is an approximation which ignores exact prostate morphology [3], therefore more accurate volume estimation methods are sought. Computer-aided diagnosis (CAD) systems that use mpMRI for prostate volume estimation and CSPCa lesion detection and/or segmentation may provide the desired performance improvements over current clinical practice.

Automatic segmentation of the prostate may enable accurate prostate volume estimation. Several automatic methods for prostate segmentation have been published [5,6,7,8,9,10]. Foremost, the PROMISE12 Challenge has driven consistent improvements in the performance of prostate segmentation algorithms over the past decade [11]; an unpublished deep learning method named MSD-Net currently tops the leader board with a mean Dice coefficient of 0.92 for whole-prostate segmentation. To the best of our knowledge, only the work by Lee et al. [10] has compared prostate volume estimation using an automatic segmentation method to the clinically utilized ellipsoid formula. On a 70-patient test set, their 3D CNN for whole-prostate segmentation achieved a mean Dice coefficient of 0.87 and a mean absolute percentage error (Abs%Err) of 11.78% for volume estimation, while the mean Abs%Err for the ellipsoid formula was 11.92%. In the discussion section of their paper, Lee et al. mention the potential benefit of more accurate volume estimation methods on the calculation of PSAd, but their study stopped short of providing a quantitative comparison.

CAD systems for lesion detection and segmentation are actively being investigated, as demonstrated by a vast and growing literature [12,13,14,15,16,17,18]. The studies by Cao et al. [12] and Schelb et al. [14] directly compared CAD systems for CSPCa lesion detection against radiologist mpMRI assessment. Cao et al. showed that their proposed FocalNet convolutional neural network (CNN), trained using biparametric MRI (bpMRI), had a CSPCa lesion detection sensitivity of 87.9%, which was only 1.5% lower than PI-RADS v2 scoring by three experienced radiologists who read a subset of cases each. Their result was obtained from a fivefold cross-validation of 417 preoperative patients who later underwent radical prostatectomy. Similarly, the study by Schelb et al. showed that a U-Net CNN [19] produced similar CSPCa detection performance to PI-RADS v2 scoring by eight radiologists who each read a subset of cases. On the held-out test cohort of 62 men sampled from the same study cohort as the training data, their method achieved a patient-level sensitivity of 92% and specificity of 47%, while radiologist assessment yielded a sensitivity of 88% and a specificity of 50%; differences in sensitivity and specificity between the proposed CNN approach and radiologist scoring were not statistically significant. While the studies by Cao et al. and Schelb et al. evaluated CAD systems using test data sampled from the same study cohort as the training data, the study by Thon et al. [15] evaluated the commercially available Watson Elementary^TM^ system using an external test cohort of 79 men. Their study concluded that Watson Elementary^TM^ did not perform satisfactorily on external test data due to differences in the instrumentation and acquisition parameters used to collect training and test data. Moreover, they remarked that optimistic performances of CAD systems reported in other studies may be dataset-specific, and therefore advocated for the necessity of external validation of CAD systems.

This work has two aims. The first aim is to introduce AutoProstate: a deep learning-powered framework for automatic MRI-based prostate cancer detection and assessment that we have developed. In particular, AutoProstate segments the prostatic zones on T2-weighted imaging (T2WI), detects and segments CSPCa lesions using bpMRI, and generates a novel automatic web-based report containing four sections: *Patient Details*, *Prostate Size and PSA Density*, *Clinically Significant Lesion Candidates*, and *Findings Summary*, which posits it close to clinical deployment. Notably, AutoProstate uses up-to-date deep learning techniques for training and inference, such as hybrid losses [20], test-time dropout [21], test-time augmentation [22], and model ensembling, to enhance performance. The second aim of this work is to perform a high-quality single-centre external validation of AutoProstate, as a first step towards clinical deployment, ahead of multicentre external validation and prospective validation in a clinical setting. In our experiment, AutoProstate is trained using the publicly available PROSTATEx dataset [23], and externally validated using the Prostate Imaging Compared to Transperineal Ultrasound-guided biopsy for significant prostate cancer Risk Evaluation (PICTURE) trial dataset [24]. The external validation follows the key considerations for authors, reviewers, and readers of AI Manuscripts in radiology by Bluemke et al. [25]. In particular, the external test set contains MRIs acquired using scanners manufactured by a different vendor to the scanners used to acquire the training set and is confirmed using transperineal template prostate-mapping (TTPM) biopsy, which avoids the biases associated with MR-guided targeted biopsy and prostatectomy [24]. Furthermore, we compare the performance of AutoProstate to the performance of an experienced radiologist who, at the time of the PICTURE trial, had 10 years’ experience in reading prostate mpMRI.

## 2. Methods

**Figure 1:**
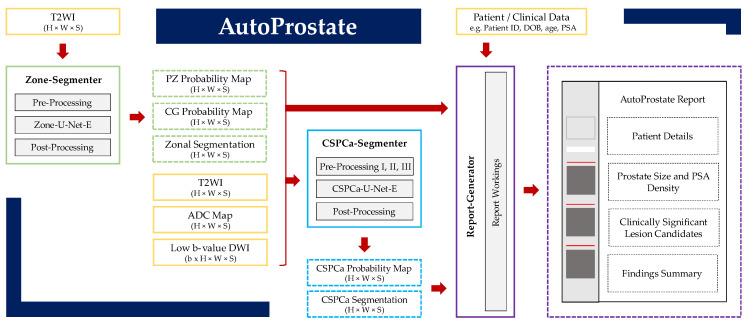
AutoProstate framework diagram. AutoProstate consists of three modules: Zone-Segmenter (green), CSPCa-Segmenter (blue), and Report-Generator (purple); solid boxes correspond to module computations, while dashed boxes correspond to module outputs. Yellow boxes indicate AutoProstate inputs from external sources.

AutoProstate, visualized schematically in Figure 1, consists of three modules: Zone-Segmenter, CSPCa-Segmenter, and Report-Generator. Methodological aspects of each module are described in detail in the subsections to follow, while specific experimental parameters used to collect results are described in Section 3.

### 2.1. Zone-Segmenter Module

The Zone-Segmenter module segments peripheral zone (PZ), central gland (CG), and background tissues on T2WI.

#### 2.1.1. Pre-Processing

T2W images are first resampled to a common in-plane resolution and cropped to a common in-plane shape, and then normalized by whitening of image voxel intensities.

#### 2.1.2. Zone-U-Net-E

After pre-processing, each T2WI slice is segmented by an ensemble of 2D nnU-Nets with task-specific hyperparameter modifications; we refer to each constituent 2D nnU-Net as Zone-U-Net and the ensemble of Zone-U-Nets as Zone-U-Net-E. A detailed description of the Zone-U-Net architecture is given in Supplementary Section S1. The output of each Zone-U-Net is slice-wise PZ, CG, and background probability maps. Per-voxel averaging is used to combine the probability map outputs of each Zone-U-Net ∈ Zone-U-Net-E, followed by restacking of slices to form PZ, CG, and background probability map volumes.

#### 2.1.3. Post-Processing

The PZ, CG, and background probability maps output by Zone-U-Net-E are transformed to the original T2WI shape and voxel resolution using padding and resampling operations. As a final step, a zonal segmentation map is obtained from the PZ, CG, and background probability maps using a per-voxel argmax operation.

### 2.2. CSPCa-Segmenter Module

The CSPCa-Segmenter module detects and segments CSPCa lesions using each patient’s T2WI, apparent diffusion coefficient (ADC) map, low b-value diffusion-weighted imaging (DWI), and PZ and CG probability maps output by Zone-Segmenter.

#### 2.2.1. Pre-Processing I: Computed High b-Value DWI

AutoProstate generates computed high b-value DWI from available DWI corresponding to low b-values (typically b ∈[0, 1000] s/mm^2^ [26]) using a monoexponential model for the per-voxel observed signal [27]:

(1) s(b)=s(0)·exp(−b ·ADC).

Using non-linear least squares to fit Equation (1) to voxel intensities belonging to low b-value images, estimates $s^{*}\left(0\right)$ of s(0) and ${\mathrm{ADC}}^{*}$ of ADC, are obtained. Subsequently, a computed high b-value image is generated using the equation:

(2) $$s\left(b_{c}\right)={\;s}^{*}\left(0\right)\cdot \exp\left(-b\;\cdot {\mathrm{ADC}}^{*}\right),$$

where $b_{c}$ is the high b-value being extrapolated.

#### 2.2.2. Pre-Processing II: Registration

Image registration is used to align ADC maps and computed high b-value DWI to T2WI to account for voluntary/involuntary patient movement between T2WI and DWI acquisitions and differences in resolution. First, ADC maps are affinely registered to T2WI using the symmetric block matching algorithm [28]. Next, a non-rigid registration is applied to the transformed ADC map using the free-form deformation (FFD) algorithm [29], with the convolution-based fast local normalized correlation coefficient (LNCC) similarity measure to enable robustness to bias field inhomogeneity [30]. Finally, the transformation obtained from the composition of both types of registration is used to register computed high b-value DWI to T2WI.

#### 2.2.3. Pre-Processing III: Resampling, Cropping, and Normalization

T2WI, registered ADC map and computed high b-value DWI, and PZ and CG probability maps are resampled to a common in-plane resolution and cropped to a common in-plane shape, centred on the prostate; image cropping is used for memory efficiency. Then, T2WI and computed high b-value DWI are normalized by dividing voxel intensities by the interquartile mean of CG voxel intensities. Our approach is a modification of the normalization approach suggested by Bonekamp et al. [31], where voxel intensities were divided by the mean of PZ voxel intensities. We opt for normalization using CG voxel intensities since CG segmentations are typically more reliable than PZ segmentations [32], and we opt for the interquartile mean of CG voxel intensities as opposed to the mean of all CG voxel intensities, to remove extremes that may correspond to abnormalities unique to a patient. ADC maps were not normalized as they contain a quantitative measurement.

#### 2.2.4. CSPCa-U-Net-E

After pre-processing, each slice of a patient’s T2WI, ADC map, computed high b-value DWI, and PZ and CG probability maps are input channel-wise to an ensemble of 2D nnU-Nets for CSPCa lesion segmentation; the addition of PZ and CG guidance as input has been shown to increase CSPCa lesion detection performance as the occurrence and appearance of prostate cancer is dependent on its zonal location [33]. We refer to each constituent 2D nnU-Net as CSPCa-U-Net and the ensemble of CSPCa-U-Nets as CSPCa-U-Net-E. A detailed description of the CSPCa-U-Net architecture is given in Supplementary Section S2. In each CSPCa-U-Net, we model epistemic uncertainty using test-time dropout, following the approach in Kendall et al. [34], i.e., dropout layers are inserted after the central three encoder units and two decoder units, with dropout probability equal to P. We model aleatoric uncertainty using test-time augmentation as in Wang et al. [22].

The output of each CSPCa-U-Net is slice-wise CSPCa probability maps. Per-voxel averaging is used to combine the probability map outputs of each CSPCa-U-Net ∈ CSPCa-U-Net-E, followed by restacking of slices to form a probability map volume.

#### 2.2.5. Post-Processing

The CSPCa probability map output by CSPCa-U-Net-E is transformed to the original T2WI shape and voxel resolution using padding and resampling operations. Next, probabilities are calibrated using an isotonic regression calibration module [35], to allow more interpretable CSPCa likelihoods. CSPCa lesion segmentations are obtained by thresholding CSPCa probability maps using a cut-off value C; C is chosen during experimentation using training data to match AutoProstate’s detection sensitivity and specificity to that of an experienced radiologist. Finally, a false-positive reduction step is applied to remove connected components smaller than MinSize mm^2^.

### 2.3. Report-Generator Module

**Figure 2:**
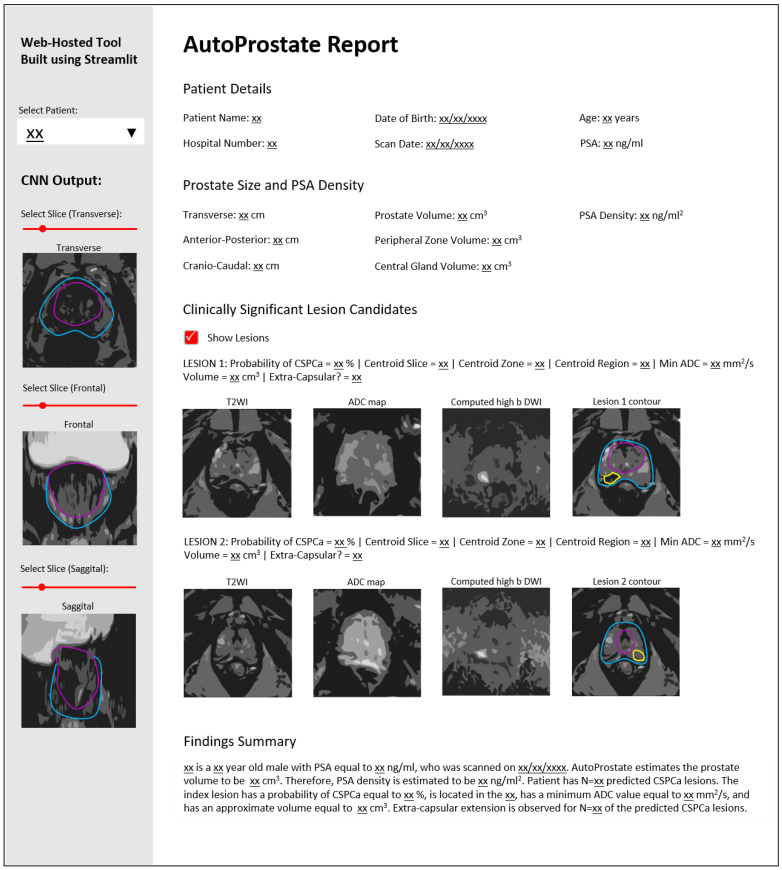
AutoProstate Report template, where xx denotes an automatically populated field.

The Report-Generator module generates an automatic report using input bpMRI and clinical data, and the outputs of the Zone-Segmenter and CSPCa-Segmenter modules; the report template is shown in Figure 2.

The left-hand pane contains interactive report elements including a patient selector and transverse, frontal, and sagittal views of zone and CSPCa lesion segmentation outputs overlaid on T2WI, with associated widgets for slice selection.

The topmost section of the main report interface is named *Patient Details*. This section includes *Patient Name*, *Hospital Number*, *Date of Birth*, *Scan Date*, *Age* (years), and *PSA* (ng/mL).

The second report section is named *Prostate Size and PSA Density*. This section presents calculated prostate lengths and volumes, and the PSAd. The *Transverse*, *Anterior–Posterior*, and *Cranio–Caudal* lengths of the prostate, in cm, are calculated using the maximum extents of the prostate on the whole-prostate segmentation, where the whole-prostate segmentation is the union of the PZ and CG segmentations. *Prostate Volume*, *Peripheral Zone Volume*, and *Central Gland Volume*, in cm^3^, are calculated by multiplying voxel counts by voxel volume. The *PSA Density* (ng/mL^2^) is calculated by dividing PSA by the calculated whole-prostate volume.

The third report section is named *Clinically Significant Lesion Candidates*. This section presents a listing of all detected CSPCa lesions, sorted in descending order of *Probability of CSPCa*. The *Centroid Slice*, *Centroid Zone* (PZ or CG), and *Centroid Region* (base, midgland, or apex) are determined based on the location of the lesion centroid; our region determination follows the methodology outlined by Litjens et al. [11] for evaluating the PROMISE12 Challenge, where the apex is defined as the caudal-most third of the prostate, the base is the cranio-most third of the prostate, and the midgland is the remaining portion. The *Min ADC* (mm^2^/s) is calculated as the minimum ADC value inside the predicted CSPCa lesion contour. As in the *Prostate Size and PSA Density* report section, *Volume* (cm^3^) is calculated by multiplying voxel counts by voxel volume. Finally, the flag *Extra-Capsular?* is set to true if the lesion contour protrudes beyond the whole-prostate contour, otherwise it is set to false.

The last section of the report is named *Findings Summary*, where key information (denoted xx in Figure 2) from other report sections is used to populate a template paragraph.

Following patient selection, the report is built using Streamlit (version 0.75.0; Available online: https://streamlit.io (accessed on 21 January 2021). Streamlit is an open-source Python library for creating shareable interactive web applications.

## 3. Experimental Setup

In this section, we describe the datasets used for training and testing AutoProstate, the methodological settings employed, and the evaluation measures used to assess performance.

### 3.1. Patient Datasets

AutoProstate was trained using the publicly available PROSTATEx dataset [23], and externally validated using the Prostate Imaging Compared to Transperineal Ultrasound-guided biopsy for significant prostate cancer Risk Evaluation (PICTURE) study dataset [24].

#### 3.1.1. PROSTATEx Dataset

Details of the PROSTATEx dataset have previously been reported [36]. A total of 346 consecutive patient studies were downloaded from the PROSTATEx Challenges database [23]. The database features mpMRI for men examined at Radboud University Medical Center between 2011 and 2012.

MpMRI was acquired using two 3-Tesla magnetic field scanners (Magnetom Trio and Skyra, Siemens) and a pelvic-phased array coil. Sequences collected included T2WI, ADC map computed from DWI acquired at multiple b-values (50, 400, 800), and DCEI with a temporal resolution of 3.5 s. All mpMRI studies were reported by an experienced radiologist with over 20 years’ experience in reading prostate mpMRI, who highlighted areas of suspicion per modality with a point marker and scored them using PI-RADS v1. MR-guided targeted biopsies of marked points with PI-RADS v1 score ≥ 3 were performed, while marked points with PI-RADS v1 score 3 (unlikely for CSPCa) were not biopsied and assumed to be clinically insignificant (5% incidence of CSPCa in PI-RADS v1 3 lesions at Radboud University Medical Center). Subsequently, biopsy specimens were graded by a histopathologist. The marked point coordinate and a ground-truth label (clinically significant equal to true or false) for each marked lesion was released publicly for 204 of the 346 patients, hence only these 204 patients feature in our work; clinical and histopathological characteristics are shown in Table S1.

Whole-prostate, zonal, and lesion contours for the 204 patients were performed by an external group [37]. In summary, contours were produced in consensus by radiology residents (2 years’ experience in reading prostate mpMRI) and board-certified radiologists (5 years’ experience in reading prostate mpMRI) at the University of Naples. Radiology residents and board-certified radiologists worked in pairs for quality control and annotation. Whole-prostate and zonal contours (PZ and CG) were drawn for each patient. In addition, 299 lesions were delineated, including 76 CSPCa lesions and 223 low-grade or benign lesions (nCSPCa).

#### 3.1.2. PICTURE Dataset

Full details of the PICTURE study have previously been reported [24,38]. Men were examined at University College London Hospital between 2012 and 2014. Inclusion criteria for the PICTURE study were: (i) men who had undergone an initial standard transrectal ultrasound-guided (TRUS) biopsy, but concern remained over the accuracy of the subsequent diagnosis; and (ii) men suitable for further characterization using transperineal template prostate-mapping (TTPM) biopsy. Exclusion criteria were: (i) previous history of prostate cancer treatment; and (ii) lack of complete gland sampling or inadequate sampling density at TTPM.

MpMRI was acquired using a 3-Tesla magnetic field scanner (Achieva, Philips Healthcare) and a pelvic-phased array coil. Sequences collected included T2WI, DWI with high b-value (2000), ADC map computed from DWI acquired at multiple b-values (0, 150, 500, 1000), and DCEI with a temporal resolution of 13 s.

All mpMRI studies were reported by an experienced radiologist with 10 years’ experience in reading prostate mpMRI, using a five-point Likert impression scale for the likelihood of CSPCa [39]; CSPCa was defined as Gleason score ≥ 3 + 4. Scoring was completed at the lesion, sector, and patient-levels. Clinical information, including the referral PSA (ng/mL), was available to the radiologist during scoring to reflect clinical practice. Men underwent MR-guided targeted biopsy of focal index lesions and TTPM biopsy with 5 mm sampling as the reference standard. TTPM biopsy was used to overcome the inaccuracies of TRUS biopsy [1] and the selection bias towards men with aggressive disease associated to radical prostatectomy [40]. Altogether, 249 men completed mpMRI and TTPM biopsy.

In this work, two patients were removed due to missing MRI data. Clinical and histopathological characteristics for the 247 included patients are shown in Table S2.

Whole-prostate and zonal contours were drawn by a board-certified radiologist (E.W.J., 3 years’ experience in the quantitative analysis of prostate mpMRI), for 80 patients. Lesions were delineated by two board-certified radiologists (S.S. and N.G., 5 and 4-years’ experience in scoring prostate mpMRI using Likert assessment and PI-RADS v2, respectively), who drew contours on a subset of cases each. The protocol for lesion contouring was agreed between the radiologists beforehand. First, histopathology reports from MR-guided targeted and TTPM biopsies were reviewed alongside mpMRI to locate the highest Gleason grade focal lesion; if there were multiple focal lesions with the maximum Gleason grade, the highest scoring focal lesion according to Likert or PI-RADS v2 was identified. Next, a single axial T2WI slice was selected corresponding to the centre of the identified lesion. Then, all focal lesions on the selected slice were contoured. Additionally, focal benign lesions that were scored Likert or PI-RADS v2 ≥ 4 were contoured in patients that were biopsy-negative for cancer. A total of 210 lesions were delineated, including 147 CSPCa lesions and 63 nCSPCa lesions.

### 3.2. Methodological Settings

In this section, we describe the training and inference settings used for conducting experiments with AutoProstate.

#### 3.2.1. Zone-Segmenter Module

T2WI were resampled to a common in-plane resolution of 0.4018 mm × 0.4018 mm and cropped to a common in-plane shape of 320 × 320.

A tenfold cross-validation analysis of Zone-U-Net was conducted using the PROSTATEx dataset to optimize training hyperparameters, loss function, and augmentations. Fold splits are shown in Table S3. Zone-U-Net performed optimally when trained for 50 epochs with learning rate equal to 0.0001, batch size equal to eight, Adam optimization [41], an equally-weighted hybrid loss composed of Dice loss [8] and Focal loss [20], and horizontal flip (probability = 0.5), rotation (−20°, 20°), and scaling (−10%, 20%) augmentations.

Following the tenfold cross-validation, the ten trained Zone-U-Nets were used to construct Zone-U-Net-E; cross-validation ensembles have been shown to be an effective ensembling strategy [32].

#### 3.2.2. CSPCa-Segmenter Module

A high b-value, $b_{c\;}=2000$, was selected for computing high b-value DWI as in Verma et al. [26].

The registration of ADC maps to T2WI employed default parameters for affine registration via symmetric block-matching. The subsequent non-rigid FFD registration used a Gaussian kernel with standard deviation equal to 5 mm for LNCC calculation, control point spacing equal to 10 mm, and bending energy constraint equal to 0.1. Registrations were run using NiftyReg (version 1.3; Available online: https://github.com/KCL-BMEIS/niftyreg (accessed on 1 October 2018). Through visual inspection, satisfactory registration was observed for the majority of PROSTATEx and PICTURE dataset cases. No manual steps were taken to correct any instances of misregistration, and cases with misregistration were not excluded from our analysis.

T2WI, registered ADC maps and computed b2000 (Cb2000) DWI, and PZ and CG probability maps, were resampled to a common in-plane resolution of 0.4018 mm × 0.4018 mm and cropped to a common in-plane shape of 256 × 256, centred on the prostate.

Like Zone-U-Net, the training settings for CSPCa-U-Net were determined through tenfold cross-validation using the PROSTATEx dataset with the fold splits shown in Table S3. CSPCa-U-Net performed optimally when trained for 50 epochs with learning rate equal to 0.0001, batch size equal to 12, Adam optimization, a dropout probability of P =0.2 for central dropout, a hybrid loss composed of the sum of Dice loss multiplied by 0.5 and Focal loss multiplied by 1.0, and horizontal flip (probability = 0.5), rotation (−20°, 20°), and scaling (−10%, +20%) augmentations. The same dropout probability and augmentation settings were used for test-time dropout and test-time augmentation.

CSPCa probability maps output by CSPCa-U-Net for each fold were calibrated using separate isotonic calibration modules for each fold. Following calibration, CSPCa probability maps were thresholded using cut-off values determined for each fold, corresponding to a lesion-level sensitivity of 93% and specificity of 37%, in the fold’s training set. The aforementioned sensitivity and specificity correspond to reference radiologist performance at PI-RADS v1 cut-off ≥ 4 on a separate patient cohort from Radboud Medical Center, reported on in Litjens et al. [42], which was used since prospective radiologist performance was not available for the PROSTATEx dataset. As a final post-processing step, connected components smaller than 40 mm^3^ were removed. UK National Institute for Health and Care Excellence (NICE) guidelines recommend a minimum size of 200 mm^3^ for CSPCa lesions [43]; we picked a minimum size of 40 mm^3^ (20% of 200 mm^3^) considering some CSPCa lesions may only be partially segmented.

Following the tenfold cross-validation, the ten trained CSPCa-U-Nets were used to construct CSPCa-U-Net-E. CSPCa-U-Net-E was calibrated using isotonic calibration. For thresholding, a cut-off value C = 4.5% was determined to match radiologist performance in the training set for CSPCa-U-Net-E i.e., the entire PROSTATEx dataset. For false-positive reduction, connected components smaller than 40 mm^3^ were removed, as in the cross-validation analysis.

### 3.3. AutoProstate External Validation Evaluation Measures

Whole-prostate and zonal segmentations were evaluated using the Dice coefficient. Prostate size measurements (transverse, anterior–posterior, and cranio–caudal lengths), as well as whole-prostate and zonal volumes, were evaluated using the Abs%Err; the ground-truth lengths and volumes used in the calculation of Abs%Err were derived from the manually-drawn whole-prostate and zonal contours. The PSAd estimated by AutoProstate was evaluated using absolute error (AbsErr), since the absolute value of PSAd has a meaning relative to risk definitions [43]; the ground-truth PSAd value used in the calculation of AbsErr was obtained by dividing PSA by the whole-prostate volume calculated using the manually-drawn whole-prostate contour. The aforementioned evaluation metrics were calculated over the 80 patients from the PICTURE dataset for which manually drawn whole-prostate and zonal segmentations were available.

Receiver operating characteristic (ROC) area under the curve (AUC) and precision-recall (PR) AUC were calculated to quantify AutoProstate’s ability to differentiate between CSPCa lesions and nCSPCa lesions. After thresholding and false-positive reduction, sensitivity, specificity, and precision were calculated at lesion-level and average false positives were calculated at patient-level. For the PICTURE dataset, the calculation of average false positives was made using 93 patients who were biopsy-negative for CSPCa, due to limitations in the ground-truth prohibiting false-positive determination in biopsy positive patients. In addition, CSPCa lesion Dice and Abs%Err of lesion area were calculated on slices containing a contour.

Prostate volume, PSAd, and lesion detection metrics computed for AutoProstate were compared to the same metrics calculated for an experienced radiologist (S.P., 10 years’ experience in scoring prostate mpMRI) who prospectively filled out a case report for each patient. Prostate volume was estimated using the ellipsoid formula and lesions were scored using a five-point Likert scale [39]. Statistical tests were used to compare the performances of AutoProstate and the experienced radiologist. The Wilcoxon’s signed-rank test [44] was used to statistically compare prostate volume and PSAd estimates, DeLong’s test [45] was used to statistically compare lesion ROC AUC, McNemar’s test [46] was used to statistically compare sensitivity and specificity, the weighted generalized score (WGS) test statistic [47] was used to statistically compare precision, and Wilcoxon’s signed-rank test was used to statistically compare average false positives.

## 4. Results

AutoProstate, trained using the PROSTATEx dataset, was externally validated using the PICTURE dataset. This section presents the results of the cross-validation of Zone-U-Net and CSPCa-U-Net (the building blocks of AutoProstate) and a detailed analysis of the external validation of AutoProstate using the PICTURE dataset, with comparisons made to the performance of an experienced radiologist with 10 years’ experience in reading prostate mpMRI, where possible.

### 4.1. Zone-U-Net and CSPCa-U-Net Tenfold Cross-Validation

Using the settings described in Section 3.2.1., Zone-U-Net achieved mean Dice coefficients of 0.78, 0.86, and 0.91 for PZ, CG, and whole-prostate segmentation, respectively.

Using the settings described in Section 3.2.2., CSPCa-U-Net achieved a mean ROC AUC of 0.85 and a mean PR AUC of 0.70. After thresholding, CSPCa-U-Net achieved a mean sensitivity of 93%, a mean specificity of 37%, a mean precision of 34%, and a mean false-positive count per patient of 6.9. Following false-positive reduction, mean sensitivity dropped marginally to 92%, mean specificity increased to 46%, mean precision increased to 37%, and mean false positives per patient dropped significantly to 3.3 (*p* 0.01). Furthermore, CSPCa-U-Net achieved a mean Dice coefficient of 0.39 for CSPCa lesion segmentation.

### 4.2. AutoProstate External Validation Analysis: Whole-Prostate and Zonal Segmentations, Prostate Size Measurements, and PSA Density

**Figure 3:**
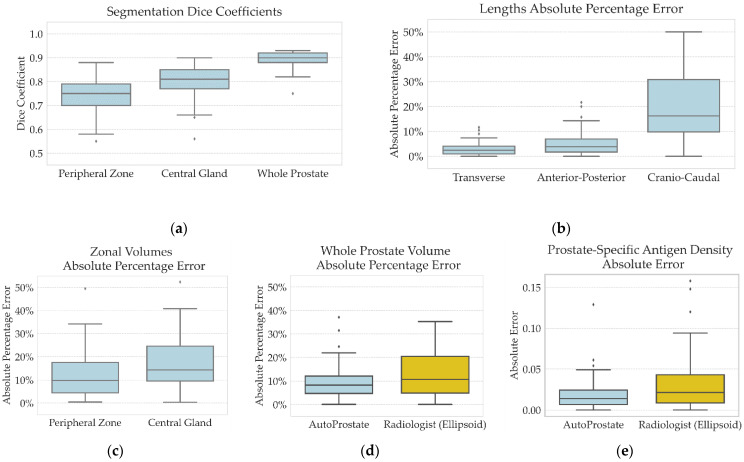
AutoProstate external validation analysis of whole-prostate and zonal segmentations, prostate size measurements, and PSAd, using 80 patients from the PICTURE dataset for which ground-truth segmentations were available: (**a**) Distribution of Dice coefficients for PZ, CG, and whole-prostate segmentation; (**b**) Distribution of Abs%Err for transverse, anterior–posterior, and cranio–caudal lengths; (**c**) Distribution of Abs%Err for PZ and CG volumes; (**d**) Distribution of Abs%Err for whole-prostate volume estimations by AutoProstate and the experienced radiologist; and (**e**) Distribution of AbsErr for PSAd calculated by AutoProstate and the experienced radiologist; the ground-truth PSAd value used to compute the AbsErr for AutoProstate and the experienced radiologist was calculated by dividing PSA by the whole-prostate volume derived from the ground-truth whole-prostate segmentation.

Table 1 and Figure 3 present summaries of the distribution of Dice coefficients for whole-prostate and zonal segmentations, the distribution of Abs%Err for prostate size measurements, and the distribution of AbsErr for PSAd calculation, for 80 patients from the PICTURE dataset for which ground-truth segmentations were available.

Mean Dice coefficients of 0.75, 0.80, and 0.89 were obtained for the PZ, CG, and whole-prostate, respectively. AutoProstate’s Zone-Segmenter module found PZ segmentation a more difficult task than CG segmentation, while whole-prostate segmentation had a higher mean Dice coefficient than both zonal segmentations, suggesting an ease of distinguishing prostate tissue from background tissues, but a difficulty in distinguishing between PZ and CG tissue. As expected, the mean Dice coefficients for the PZ, CG, and whole-prostate segmentations were lower than those obtained on the PROSTATEx dataset during the tenfold cross-validation of Zone-U-Net (0.78, 0.86, and 0.91 for PZ, CG, and whole-prostate segmentation, respectively) which may be indicative of a generalization gap due to acquisition/population differences.

The transverse, anterior–posterior, and cranio–caudal lengths of the prostate were estimated using the whole-prostate segmentation output by Zone-Segmenter. Mean Abs%Err of 3%, 5%, and 20% were obtained for transverse, anterior–posterior, and cranio–caudal lengths, respectively. In addition to the lowest mean Abs%Err, the transverse length had a smaller standard deviation than anterior–posterior and cranio–caudal lengths. Through visual inspection of segmentation outputs, we attribute the variability in the accuracy of the anterior–posterior measurement to the difficulty of determining prostate extent in the anterior fibromuscular stroma, and similarly, we attribute the variability in the accuracy of the cranio–caudal measurement to the difficulty of determining prostate extent at the base and apex regions of the prostate. Strikingly, a large maximum Abs%Err of 100% was observed for the cranio–caudal measurement, due to under-segmentation of the base region in the ground-truth.

PZ, CG, and whole-prostate volumes were calculated using the PZ, CG, and whole-prostate segmentations output by Zone-Segmenter. Mean Abs%Errs of 12%, 18%, and 9% were obtained for PZ, CG, and whole-prostate volumes, respectively. Strikingly, a large maximum Abs%Err of 112% was observed for the CG, which was found to be due to over-segmentation of the CG in the base region.

We compare the Abs%Err of the whole-prostate volume calculated by AutoProstate to the same calculated by the experienced radiologist who used the ellipsoid formula, which is clinically advocated. AutoProstate had a mean Abs%Err of 9%, while the experienced radiologist’s mean Abs%Err was 13%; the difference was statistically significant (*p* 0.05). Using the whole-prostate volumes computed by AutoProstate and the experienced radiologist, PSAd was calculated. AutoProstate achieved a mean AbsErr of 0.019, while the experienced radiologist’s mean AbsErr was 0.031; again, the difference was statistically significant (*p* 0.05).

### 4.3. AutoProstate External Validation Analysis: Clinically Significant Prostate Cancer Lesion Detection and Segmentation

**Figure 4:**
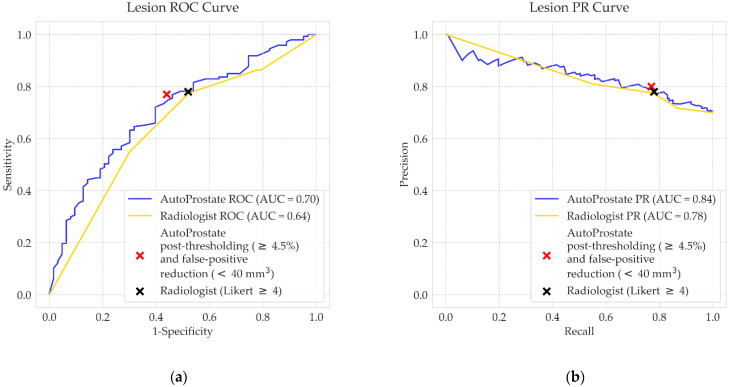
PICTURE dataset CSPCa lesion detection (**a**) ROC curves and (**b**) PR curves, corresponding to the experienced radiologist (gold) and AutoProstate (blue).

CSPCa lesion detection performance for AutoProstate and the experienced radiologist are shown in Table 2, while Figure 4 shows the ROC and PR curves for AutoProstate and the radiologist.

AutoProstate achieved a mean ROC AUC of 0.70 and a mean PR AUC of 0.84, calculated using output CSPCa probability maps prior to thresholding. After thresholding the CSPCa probability maps using a cut-off value equal to 4.5%, the following were obtained: a sensitivity of 78%, a specificity of 49%, a precision of 78%, and a mean false-positive count of 6.1. Following false-positive reduction, mean sensitivity dropped marginally to 76%, mean specificity increased to 57%, mean precision increased marginally to 80%, and the mean false-positive count per patient dropped to 2.5.

Likert scores assigned to suspicious lesions by the experienced radiologist were used to calculate ROC and PR curves; radiologist Likert scoring gave a ROC AUC of 0.64 and PR AUC of 0.78. After thresholding at cut-off score Likert ≥ 4, the following were obtained: a sensitivity of 78%, a specificity of 48%, a precision of 78%, and a mean false-positive count of 0.3. Differences between the ROC AUC, PR AUC, sensitivity, specificity, and precision of AutoProstate and the experienced radiologist were not statistically significant. However, the difference between mean false positives was statistically significant (*p* 0.001).

A further analysis was completed to assess the level of agreement between AutoProstate and the experienced radiologist’s Likert scores, on annotated lesions, as shown in Table S4. For CSPCa lesions, there was a 78% (114/147) concordance between AutoProstate and the experienced radiologist, while for nCSPCa lesions, there was a 62% (39/63) concordance.

**Figure 5:**
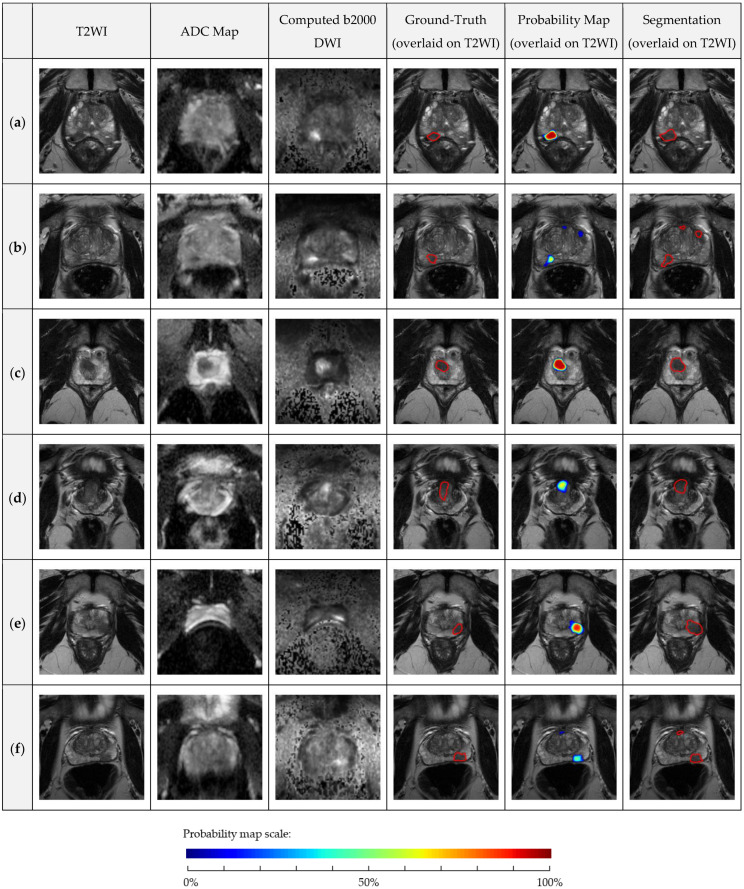
PICTURE dataset axial T2WI, ADC map, Cb2000 DWI, ground-truth lesion contour overlaid on T2WI, probability map overlaid on T2WI, and segmentation overlaid on T2WI: (a) 79-year-old man, PSA 12.57 ng/mL, midgland PZ GS 4 + 3 lesion, Likert 5, AutoProstate probability of CSPCa 100%; (b) 66-year-old man, PSA 7.50 ng/mL, midgland PZ GS 3 + 4 lesion, Likert 3, AutoProstate probability of CSPCa 65%; (c) 64-year-old man, PSA 10.53 ng/mL, apex CG GS 3 + 4 lesion, Likert 5, AutoProstate probability of CSPCa 95%; (d) 56-year-old man, PSA 7.91 ng/mL, base CG GS 3 + 4 lesion, Likert 4, AutoProstate probability of CSPCa 66%; (e) 60-year-old man with stable rectal gas-induced magnetic susceptibility artefact on DWI, PSA 6.15 ng/mL, midgland PZ GS 3 + 4 lesion, Likert 5, AutoProstate probability of CSPCa 88%; and (f) 73-year-old man with bowel peristalsis-induced magnetic susceptibility artifact, PSA 4.09 ng/mL, midgland PZ GS 3 + 4 lesion, Likert 5, AutoProstate probability of CSPCa 49%.

**Figure 6:**
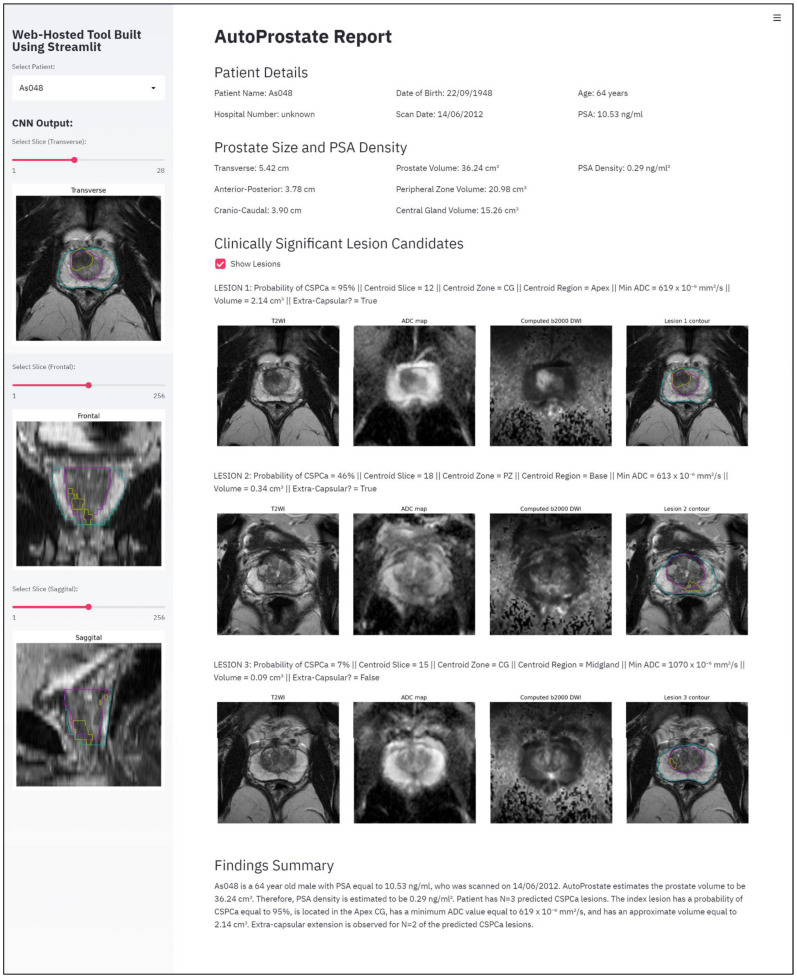
AutoProstate Report for a 64-year-old man with PSA equal to 10.53 ng/mL who participated in the PICTURE study. LESION 1 (probability of CSPCa equal to 95%) corresponds to a biopsy-proven GS 3+4 lesion, while LESION 2 and LESION 3 (probabilities of CSPCa equal to 46% and 7%, respectively) are false positives.

AutoProstate’s lesion segmentations enable the calculation of lesion volume and lesion minimum ADC. Lesion segmentation accuracy, evaluated using the Dice coefficient, was calculated using slices containing a corresponding ground-truth CSPCa lesion contour. The following Dice coefficient metrics were obtained: a mean of 0.46 (SD: 0.32), a median of 0.58 (IQR: 0.10–0.72), and a min–max range of 0.00–0.90. Several example CSPCa lesion segmentations are presented in Figure 5. Examples are shown in the PZ and CG, and in the base, midgland, and apex regions of the prostate. In addition, examples have been included to demonstrate AutoProstate’s robustness to magnetic susceptibility artifacts. Furthermore, an example automatic report generated by AutoProstate is shown in Figure 6.

## 5. Discussion

In this work, we introduced AutoProstate, a deep learning-powered framework for automatic MRI-based prostate cancer assessment. AutoProstate consists of three modules: Zone-Segmenter, CSPCa-Segmenter, and Report-Generator. The output of AutoProstate is an automatic web-based report that presents patient details, prostate size measurements and PSAd, a listing of candidate CSPCa lesions with derived characteristics, and a findings summary. AutoProstate, trained using the publicly available PROSTATEx dataset, was externally validated using the PICTURE dataset. During the external validation, the performance of AutoProstate was compared to the performance of an experienced radiologist with 10 years’ experience in reading prostate mpMRI, who prospectively estimated prostate volume and PSAd using the ellipsoid formula, and scored lesions using a five-point Likert scale.

PZ, CG, and whole-prostate segmentations are output by AutoProstate’s Zone-Segmenter module. During the experimental setup phase, we tested Zone-U-Net, prior to ensembling of Zone-U-Nets to form Zone-U-Net-E. Zone-U-Net achieved mean Dice coefficients of 0.78, 0.86, and 0.91 for PZ, CG, and whole-prostate segmentation, respectively, in tenfold cross-validation using the PROSTATEx dataset. Our result compares well to recent works by Aldoj et al. [6], where their proposed Dense-2 U-Net CNN was evaluated using fourfold cross-validation of a 188-patient subset from the PROSTATEx dataset, and to a recent work by Cuocolo et al. [7], where the previously proposed ENet CNN [48] was evaluated using a 105-patient test set from the PROSTATEx dataset. Aldoj et al. obtained mean Dice coefficients of 0.78, 0.91, and 0.92, and Cuocolo et al. obtained mean Dice coefficients of 0.71, 0.87, and 0.91, for PZ, CG, and whole-prostate segmentation, respectively. However, direct comparison between our work and the works of Aldoj et al. and Cuocolo et al. is not possible due to the use of different subsets of data for testing. During the external validation of AutoProstate using the PICTURE dataset, where Zone-U-Net-E was used for PZ, CG, and whole-prostate segmentation, AutoProstate achieved mean Dice coefficients of 0.75, 0.80, and 0.89, respectively, on 80 patients for which ground-truth segmentations were available. Antonelli et al. [49] previously reported segmentation results for the PICTURE dataset. A multi-atlas segmentation approach featuring a novel genetic atlas selection strategy was proposed; mean Dice coefficients of 0.72 and 0.83 were reported for PZ and CG segmentation, using leave-one-out cross-validation, and a mean Dice coefficient of 0.83 was reported for whole-prostate segmentation, using atlases from the PROMISE12 dataset [11].

An accurate whole-prostate segmentation is crucial for downstream calculations of prostate volume and PSAd [50]. AutoProstate’s estimate of prostate volume was compared to an estimate obtained using the ellipsoid formula, which is clinically advocated [51]. AutoProstate achieved a mean Abs%Err of 9%, while the radiologist computed ellipsoid formula estimate had a mean Abs%Err of 13%. Notably, the difference in mean Abs%Err was statistically significant (p=0.0051<0.05). Furthermore, we compared PSAd estimates obtained using the volume estimates; we found a mean AbsErr of 0.019 for AutoProstate and a mean AbsErr of 0.031 for the radiologist; again, the difference was statistically significant (p=0.0018<0.05). Since PSAd is used clinically to inform the decision to biopsy or to discharge patients [52] and furthermore, to monitor patients on active surveillance, as recommended by NICE guidelines in the UK [43], we believe a case exists for replacement of the ellipsoid formula with automated methods such as ours.

AutoProstate’s foremost purpose is to detect and segment CSPCa lesions. During the experimental setup phase, we tested CSPCa-U-Net, prior to ensembling of CSPCa-U-Nets to form CSPCa-U-Net-E. Markedly, CSPCa-U-Net achieved a lesion-level mean ROC AUC of 0.85 in tenfold cross-validation using the PROSTATEx dataset, while previous studies have reported a lesion-level mean ROC AUC of 0.81 on the same subset of PROSTATEx data used in this study, using the same input modalities. During the external validation of AutoProstate using the PICTURE dataset, where CSPCa-U-Net-E was used to segment CSPCa lesions, AutoProstate achieved a lesion-level ROC AUC of 0.70. Notably, we observed a large reduction in ROC AUC on the PICTURE dataset from that seen during the PROSTATEx dataset tenfold cross-validation. We believe that the main reason for the reduction in ROC AUC is the use of TTPM biopsy in the PICTURE study, which allowed lesions that were not prospectively identified by the radiologist, to be retrospectively contoured using TTPM biopsy findings. Other reasons may include a high occurrence of magnetic susceptibility artifacts on DWI in the PICTURE dataset and a possible generalization gap between training data and external testing data due to population/acquisition differences. On the PICTURE dataset, radiologist Likert assessment achieved a lesion-level ROC AUC of 0.64; the difference in ROC AUC between AutoProstate and the experienced radiologist was not statistically significant. Following thresholding and false-positive reduction, AutoProstate achieved a lesion-level sensitivity of 76%, a lesion-level specificity of 57%, and 2.5 false positives per patient (calculated over patients without CSPCa, only). In comparison, radiologist Likert assessment thresholded at Likert ≥ 4, achieved a lesion-level sensitivity of 78%, a lesion-level specificity of 48%, and 0.3 false positives per patient (calculated over patients without CSPCa, only); only the difference between the number of false positive detections by AutoProstate and the experienced radiologist was statistically significant (p0.001). While AutoProstate has demonstrated an ability to differentiate between CSPCa lesions and low-grade/benign lesions at the level of an experienced radiologist, further work is needed to reduce the number of false positives produced. Interestingly, AutoProstate achieved a similar sensitivity and improved specificity compared to the radiologist on annotated CSPCa and nCSPCa lesions but had a higher overall false-positive count. Therefore, it’s possible that the additional false positives produced by AutoProstate, that were not prospectively scored by the radiologist, may be easy for radiologists to rule-out.

Several aspects of this study have been guided by the set of nine key considerations for authors, reviewers, and readers of artificial intelligence studies in radiology by Bluemke et al. [25]. As recommended, we maintained a clear separation between training data and testing data. In particular, we avoided a common pitfall observed in previous studies [12,15], by determining the probability cut-off value using training data, rather than a biased approach involving the test data itself. In line with further recommendations by Bluemke et al., we were able to externally validate AutoProstate using the PICTURE dataset. Furthermore, the PICTURE dataset was acquired using Phillips’ scanners, while the PROSTATEx dataset, used to train AutoProstate, was acquired using Siemens’ scanners, meaning a further recommendation on using multivendor data for evaluation was met. Moreover, we compared AutoProstate to an expert radiologist who prospectively reported PICTURE dataset patients, and both AutoProstate and the radiologist were compared to an accepted reference standard which combined TTPM and MR-guided targeted biopsies; TTPM biopsy is highly accurate and avoids biases associated to MR-guided targeted biopsy, transrectal ultrasound-guided (TRUS) biopsy, and prostatectomy [24].

CAD system studies should describe how the CAD system will be deployed clinically, so future prospective trials can be planned accordingly. Our goal in this study was to understand the strengths, weaknesses, and idiosyncrasies of AutoProstate through a comparison against an experienced radiologist. In the clinical workflow, we envision AutoProstate as a radiologist companion system during clinical reads to allow enhanced clinical reporting. It should be acknowledged that current CAD systems for MRI-based prostate cancer diagnosis contain varying degrees of error in terms of producing too many false positives, false negatives, or both. Since the automatic report produced by AutoProstate presents visual segmentation outputs, as well as derived measurements, all outputs produced by AutoProstate can be rapidly verified by the radiologist. In particular, automatic report information deemed to be accurate can be used to prepare the patient’s clinical report, while erroneous information can be recalculated using current clinical methods or ignored if not required.

There were three limitations in our study. Firstly, our training data was limited to 76 CSPCa lesions and 223 nCSPCa lesions; we may expect improved detection sensitivity and reduced false positives if a bigger training dataset with more lesions is available. Secondly, our external validation was limited to a single external site. Thirdly, lesion contours for each PICTURE dataset patient were drawn by a single radiologist only. While the location and Gleason score of lesions was confirmed by a combination of TTPM and MR-guided targeted biopsies, we were not able to overcome the inter-reader variation known to exist in lesion boundary determination [53].

Our future work will be to perform a prospective validation of Autoprostate. In particular, we will plan a clinical trial that investigates the impact of the automatic report on the prospective clinical read of radiologists of varying levels of experience. In preparation for the prospective validation, we will seek a larger multi-centre and multi-vendor training dataset.

## 6. Conclusions

In this work, we presented AutoProstate for automatic MRI-based prostate cancer assessment. External validation using the PICTURE dataset demonstrated statistically significant improvements in prostate volume and PSA density estimation and no statistically significant differences in CSPCa lesion detection performance, when compared to an experienced radiologist with over 10 years’ experience in reading prostate mpMRI. However, further work is needed to reduce the number of false positives produced by AutoProstate, prior to prospective validation.

## Figures and Tables

**Table 1:** AutoProstate external validation analysis of whole-prostate and zonal segmentations, prostate size measurements, and PSAd, using 80 patients from the PICTURE dataset for which ground-truth segmentations were available.

Evaluation Measure	Mean (SD)	Median (IQR)	Min–Max
Experienced Radiologist ^†^			
Whole-prostate volume Abs%Err	13 (11)	11 (5–20)	0–66
PSA density AbsErr	0.031 (0.032)	0.022 (0.008–0.043)	0.000–0.158
AutoProstate			
Segmentation			
Peripheral zone Dice coefficient	0.75 (0.06)	0.75 (0.70–0.79)	0.55–0.88
Central gland Dice coefficient	0.80 (0.07)	0.81 (0.77–0.85)	0.56–0.90
Whole-prostate Dice coefficient	0.89 (0.03)	0.90 (0.88–0.92)	0.75–0.93
Lengths			
Transverse length Abs%Err	3 (2)	2 (1–4)	0–12
Anterior–posterior length Abs%Err	5 (4)	4 (2–7)	0–22
Cranio–caudal length Abs%Err	20 (15)	16 (10–31)	0–100
Volumes and PSA density			
Peripheral zone volume Abs%Err	12 (10)	10 (4–18)	0–49
Central gland volume Abs%Err	18 (15)	14 (10–25)	0–112
Whole-prostate volume Abs%Err *	9 (7)	8 (5–12)	0–37
PSA density AbsErr *	0.019 (0.020)	0.014 (0.006–0.025)	0.000–0.129

AbsErr: absolute error; Abs%Err: absolute percentage error; IQR: interquartile range; Max: maximum; Min: minimum; PSA: prostate-specific antigen; SD: standard deviation. ^†^ the experienced radiologist used the ellipsoid formula to estimate whole-prostate volume. * indicates a *p*-value 0.05 for AutoProstate compared to the experienced radiologist.

**Table 2:** PICTURE dataset CSPCa lesion detection metrics for the experienced radiologist and AutoProstate. Mean and standard deviation of false positives per patient were calculated using the 93 PICTURE dataset patients who were biopsy-negative for CSPCa, rather than over all patients, due to limitations in the ground-truth. All other metrics shown are calculated at the lesion level for the 147 CSPCa lesions and 63 nCSPCa lesions.

Experienced Radiologist (Likert Scoring)	
ROC AUC	0.64 (0.56–0.72)
PR AUC	0.78 (0.71–0.84)
Post-thresholding (cut-off: Likert ≥4)	
Sensitivity/recall (%)	78 (71–84)
Specificity (%)	48 (35–60)
Precision (%)	78 (71–84)
Mean false positives per patient	0.3 (0.2–0.4)
AutoProstate	
ROC AUC	0.70 (0.62–0.78)
PR AUC	0.84 (0.77–0.90)
Post-thresholding (cut-off: ≥4.5%)	
Sensitivity/recall (%)	78 (71–85)
Specificity (%)	49 (37–62)
Precision (%)	78 (71–85)
Mean false positives per patient *	6.1 (5.5–6.8)
Post-thresholding (cut-off: ≥4.5%) and false-positive reduction (40 mm^3^)	
Sensitivity/recall (%)	76 (68–82)
Specificity (%)	57 (45–69)
Precision (%)	80 (74–87)
Mean false positives per patient *	2.5 (2.2–2.8)

AUC: area under curve; PR: precision-recall; ROC: receiver operating characteristic. * indicates a *p*-value 0.001 for AutoProstate compared to the radiologist.

## Data Availability

PROSTATEx dataset data citation: Geert Litjens, Oscar Debats, Jelle Barentsz, Nico Karssemeijer, and Henkjan Huisman. “ProstateX Challenge data”, The Cancer Imaging Archive (2017). DOI: 10.7937/K9TCIA.2017.MURS5CL. PROSTATEx dataset masks citation: R. Cuocolo, A. Stanzione, A. Castaldo, D.R. De Lucia, M. Imbriaco, Quality control and whole-gland, zonal and lesion annotations for the PROSTATEx challenge public dataset, Eur. J. Radiol. (2021).

## Supplementary Materials

The following are available online at https://www.mdpi.com/article/10.3390/cancers13236138/s1, Section S1: Zone-U-Net Architecture, Section S2: CSPCa-U-Net Architecture, Table S1: PROSTATEx dataset characteristics, Table S2: PICTURE dataset characteristics, Table S3: Ten-fold cross-validation fold split of the PROSTATEx dataset. Lesion significance, size, and zone were used for fold stratification.
