# Supplementary material for: AutoProstate: Towards Automated Reporting of Prostate MRI for Prostate Cancer Assessment Using Deep Learning
Source: Cancers (Basel). 2021 Dec 6;13(23):6138. doi: 10.3390/cancers13236138 (PMC8656605; doi:10.3390/cancers13236138)
Supplement: Supplementary file 1 [file cancers-13-06138-s001.zip › cancers-1483077-supplementary.pdf]

cancers

MDPI

Supplementary Materials

AutoProstate: Towards Automated Reporting of Prostate MRI for Prostate Cancer Assessment using Deep Learning

Supplementary Sections

S1. Zone-U-Net Architecture

Specifically, each Zone-U-Net features six encoding blocks and five decoding blocks. Each encoding block consists of two convolutional layers with stride one 3 × 3 convolutions with zero padding, leaky rectified linear unit (LReLU) activation (neg. slope 1e−2) and instance normalization, followed by a stride two 2 × 2 max pooling operation in the first five encoding blocks. Thirty-two feature maps are output by convolutional layers in the first encoding block, with feature maps doubling in each subsequent encoding block. Upsampling deconvolution operations are used in the decoding blocks, which receive semantic information from the last encoding block and higher resolution feature maps from encoder-to-decoder skip connections.

S2. CSPCa-U-Net Architecture

CSPCa-U-Net features five encoding blocks and four decoding blocks. Each encoding block features two convolutional layers with stride one 3 × 3 convolutions with zero padding, LReLU activation (neg. slope 1e−2) and instance normalization, followed by a stride two 2 × 2 max pooling operation in the first four encoding blocks; 64 feature maps are output by convolutional layers in the first encoding block, with feature maps doubling in each subsequent encoding block. Upsampling deconvolution operations are used in the decoding blocks, which receive semantic information from the last encoder block and higher resolution feature maps from encoder-to-decoder skip connections.

Supplementary Tables

Supplementary Table S1. PROSTATEx dataset characteristics.

Center	Radboud Medical Center
Scan dates	2011 to 2012
Scanner model(s)	Siemens 3-T MAGNETOM Trio and Skyra
Reader experience	>20 years
Reporting standard	PI-RADS v1
Reference standard	MR-guided targeted biopsy of PI-RADS ≥ 3 lesions<br>PI-RADS = 2 lesions not biopsied (< 5% occurrence of CSPCa in PI-RADS = 2 lesions at center)
No. of included patients	204
Patient population	Clinically suspected
Age (years)	Not available
PSA (ng/ml)	Not available
**Per-patient maximum Gleason score**	
Not biopsied (max PI-RADS = 2, nCSPCa assumed) or only benign findings following biopsy	105
Gleason score ≤ 6	29
Gleason score 3+4	38
Gleason score 4+3	19
Gleason score 8	7
Gleason score 9-10	6
**MRI-detected lesions**	
No. of patients without MRI-detected lesions	4
No. of patients with MRI-detected lesions	200
Total annotated lesions	299
**Per-lesion Gleason score**	
Not biopsied (PI-RADS = 2, nCSPCa assumed) or benign following biopsy	187
Gleason score ≤ 6	36
Gleason score 3+4	41
Gleason score 4+3	20
Gleason score 8	8
Gleason score 9-10	7
**Per-lesion zone**	
Peripheral zone	157
Central gland	122
Both zones	20

CSPCa: clinically significant prostate cancer; MRI: magnetic resonance imaging; nCSPCa: not CSPCa; PI-RADS: Prostate Imaging-Reporting and Data System; PSA: prostate-specific antigen.

Supplementary Table S2. PICTURE dataset characteristics.

Center	University College London Hospital
Scan dates	2012 to 2014
Scanner model(s)	Philips Healthcare 3-T Achieva
Reader experience	>10 years
Reporting standard	Likert
Reference standard	TTPM biopsy (5mm sampling) + targeted biopsy
No. of included patients	247
Patient population	Clinically suspected
Median age (years)	62 (58 – 67)
Median PSA (ng/ml)	6.87 (5.09 – 9.60)
Median PSA density (ng/ml^2)	0.18 (0.14 – 0.28)
**Per-patient Likert score**	
Likert 2	22
Likert 3	82
Likert 4	49
Likert 5	94
**Per-patient maximum Gleason score**	
No prostate cancer	34
Gleason score 3+3	59
Gleason score 3+4	114
Gleason score 4+3	34
Gleason score 8	5
Gleason score 9-10	1
**MRI-detected lesions**	
No. of patients without MRI-detected lesions	66
No. of patients with MRI-detected lesions	181
Total annotated lesions	210
**Per-lesion Likert score**	
Not identified prospectively	33
Likert 2	1
Likert 3	29
Likert 4	47
Likert 5	100
**Per-lesion Gleason score**	
Benign	9
Gleason score 3+3	54
Gleason score 3+4	113
Gleason score 4+3	29
Gleason score 8	4
Gleason score 9-10	1
**Per-lesion zone**	
Peripheral zone	134
Central gland	57
Both zones	19

MRI: magnetic resonance imaging; PSA: prostate-specific antigen; TTPM: transperineal template prostate-mapping biopsy.

Supplementary Table S3. Ten-fold cross-validation fold split of the PROSTATEx dataset. Lesion significance, size, and zone were used for fold stratification.

Patient ID	Fold	Patient ID	Fold	Patient ID	Fold	Patient ID	Fold
ProstateX0000	10	ProstateX0051	4	ProstateX0102	3	ProstateX0153	8
ProstateX0001	9	ProstateX0052	1	ProstateX0103	1	ProstateX0154	6
ProstateX0002	7	ProstateX0053	2	ProstateX0104	8	ProstateX0155	10
ProstateX0003	8	ProstateX0054	5	ProstateX0105	1	ProstateX0156	5
ProstateX0004	1	ProstateX0055	8	ProstateX0106	2	ProstateX0157	10
ProstateX0005	9	ProstateX0056	2	ProstateX0107	5	ProstateX0158	2
ProstateX0006	7	ProstateX0057	4	ProstateX0108	2	ProstateX0159	8
ProstateX0007	1	ProstateX0058	6	ProstateX0109	7	ProstateX0160	9
ProstateX0008	6	ProstateX0059	2	ProstateX0110	3	ProstateX0161	3
ProstateX0009	1	ProstateX0060	4	ProstateX0111	6	ProstateX0162	6
ProstateX0010	6	ProstateX0061	6	ProstateX0112	2	ProstateX0163	9
ProstateX0011	6	ProstateX0062	7	ProstateX0113	7	ProstateX0164	2
ProstateX0012	9	ProstateX0063	10	ProstateX0114	9	ProstateX0165	10
ProstateX0013	2	ProstateX0064	4	ProstateX0115	7	ProstateX0166	1
ProstateX0014	3	ProstateX0065	5	ProstateX0116	4	ProstateX0167	3
ProstateX0015	6	ProstateX0066	1	ProstateX0117	5	ProstateX0168	2
ProstateX0016	7	ProstateX0067	6	ProstateX0118	9	ProstateX0169	4
ProstateX0017	10	ProstateX0068	3	ProstateX0119	1	ProstateX0170	3
ProstateX0018	6	ProstateX0069	10	ProstateX0120	10	ProstateX0171	2
ProstateX0019	3	ProstateX0070	2	ProstateX0121	5	ProstateX0172	1
ProstateX0020	9	ProstateX0071	4	ProstateX0122	7	ProstateX0173	3
ProstateX0021	1	ProstateX0072	9	ProstateX0123	6	ProstateX0174	9
ProstateX0022	3	ProstateX0073	9	ProstateX0124	4	ProstateX0175	8
ProstateX0023	8	ProstateX0074	3	ProstateX0125	4	ProstateX0176	7
ProstateX0024	1	ProstateX0075	1	ProstateX0126	6	ProstateX0177	4
ProstateX0025	3	ProstateX0076	5	ProstateX0127	9	ProstateX0178	4
ProstateX0026	5	ProstateX0077	8	ProstateX0128	6	ProstateX0179	2
ProstateX0027	9	ProstateX0078	5	ProstateX0129	1	ProstateX0180	5
ProstateX0028	10	ProstateX0079	7	ProstateX0130	4	ProstateX0181	2
ProstateX0029	8	ProstateX0080	4	ProstateX0131	4	ProstateX0182	3
ProstateX0030	5	ProstateX0081	5	ProstateX0132	4	ProstateX0183	2
ProstateX0031	10	ProstateX0082	3	ProstateX0133	8	ProstateX0184	7
ProstateX0032	10	ProstateX0083	7	ProstateX0134	10	ProstateX0185	8
ProstateX0033	8	ProstateX0084	2	ProstateX0135	8	ProstateX0186	4
ProstateX0034	10	ProstateX0085	6	ProstateX0136	10	ProstateX0187	5
ProstateX0035	7	ProstateX0086	1	ProstateX0137	3	ProstateX0188	8
ProstateX0036	2	ProstateX0087	7	ProstateX0138	5	ProstateX0189	1
ProstateX0037	2	ProstateX0088	7	ProstateX0139	3	ProstateX0190	5
ProstateX0038	9	ProstateX0089	5	ProstateX0140	2	ProstateX0191	4
ProstateX0039	10	ProstateX0090	6	ProstateX0141	6	ProstateX0192	8
ProstateX0040	8	ProstateX0091	8	ProstateX0142	3	ProstateX0193	4
ProstateX0041	2	ProstateX0092	10	ProstateX0143	7	ProstateX0194	10
ProstateX0042	5	ProstateX0093	3	ProstateX0144	8	ProstateX0195	7
ProstateX0043	5	ProstateX0094	8	ProstateX0145	1	ProstateX0196	3
ProstateX0044	6	ProstateX0095	9	ProstateX0146	6	ProstateX0197	10
ProstateX0045	3	ProstateX0096	1	ProstateX0147	7	ProstateX0198	1
ProstateX0046	4	ProstateX0097	8	ProstateX0148	1	ProstateX0199	9
ProstateX0047	10	ProstateX0098	4	ProstateX0149	9	ProstateX0200	9
ProstateX0048	5	ProstateX0099	4	ProstateX0150	2	ProstateX0201	7
ProstateX0049	9	ProstateX0100	9	ProstateX0151	7	ProstateX0202	3
ProstateX0050	10	ProstateX0101	5	ProstateX0152	6	ProstateX0203	1

Supplementary Table S4. Agreement and disagreement between radiologist Likert scoring and AutoProstate, on PICTURE dataset annotated lesions; grey shading indicates concordance, gold shading indicates superior performance by the experienced radiologist, and blue shading indicates superior performance by AutoProstate.

		CSPCa Lesions (n = 147)		nCSPCa Lesions (n = 63)	
		**AutoProstate**			
		Predicted nCSPCa	Predicted CSPCa	Predicted nCSPCa	Predicted CSPCa
**Radiologist**	Not identified prospectively	8% (12/147)	5% (8/147)	16% (10/63)	5% (3/63)
	Likert 2	0% (0/147)	0% (0/147)	2% (1/63)	0% (0/63)
	Likert 3	4% (6/147)	5% (7/147)	16% (10/63)	10% (6/63)
	Likert 4	7% (10/ 147)	16% (23/147)	14% (9/63)	8% (5/63)
	Likert 5	5% (8/ 147)	50% (73/147)	10% (6/63)	21% (13/63)

CSPCa: clinically significant prostate cancer; nCSPCa: not clinically significant prostate cancer.
